# Serious Complications of the Percutaneous A1 Pulley Release: Case Reports and Literature Review

**DOI:** 10.1055/a-2179-3911

**Published:** 2024-02-07

**Authors:** Dong Chul Lee, Kyung Jin Lee, Hohyung Lee, Sung Hoon Koh, Jin Soo Kim, Si Young Roh

**Affiliations:** 1 Department of Plastic and Reconstructive Surgery, Gwangmyeong Sungae General Hospital, Gwangmyeong, Republic of Korea

**Keywords:** trigger finger, percutaneous A1 pulley release, complications

## Abstract

Percutaneous first annular pulley (A1 pulley) release, which has been increasingly used to treat trigger fingers, has been widely established as a safe and simple procedure. Multiple studies have reported positive results of percutaneous A1 pulley release. In this study, however, we report cases of patients who developed complications after undergoing percutaneous A1 pulley release at local clinics. A total of six patients visited our hospital for infectious complications after percutaneous A1 pulley release. Various sequelae such as damage to normal structures, insufficient procedure, and tissue necrosis were observed during the exploration. A retrospective study was conducted to identify the cause and trend of the observed complications by instruments (HAKI knife or needle). In the HAKI knife group, there was a tendency for damage to normal structures, while in the needle group, an insufficient release or serious soft tissue necrosis was observed. Based on these cases, our findings confirm the existence and characteristics of infectious complications following the percutaneous A1 pulley release. We further identify that the type of instrument used predicts the nature of complications. Thus, reliable and skilled performance of the procedure by experts is essential for safe treatment.

## Introduction

Trigger finger is one of the most common causes of hand pain and dysfunction. It is also called stenosing tenosynovitis since it is the result of inflammation of the synovium surrounding the tendon. Trigger finger mainly manifests at the first annular pulley (A1 pulley) of frequently used fingers, followed by a subsequent narrowing and inflammation of the pulley leading to a motor disorder. As a result, malfunctions involving the gliding functions of the tendon occur, triggering flares. Initial treatment includes symptomatic treatment, nonsteroidal anti-inflammatory drugs, massage, splinting, and corticosteroid injection. 1 When these treatments do not show any improvement in the prognosis of the condition, surgical release is a certain and effective treatment. Percutaneous pulley release is a cheap, safe, and effective technique, and it is a quicker and less painful alternative with better rehabilitation results than conventional open surgical release. 2 3 Here, we report six cases of serious infectious complications, including organ injury and soft tissue necrosis, in patients who underwent percutaneous A1 pulley release.

### Percutaneous A1 Pulley Release

Trigger finger is commonly caused by the swelling and thickening of the synovium, leading to stenosis within the fibrous sheath and affecting tendon glide. The locking typically occurs at the A1 pulley. To address this issue, releasing the A1 pulley is crucial. Open trigger finger release is generally regarded as a straightforward and reliable procedure. 4

However, as a less invasive and more time-efficient alternative to the open technique, percutaneous A1 pulley release using a tenotome was initially described by Lorthioir. 5 Subsequently, Eastwood et al proposed a procedure utilizing a needle. 6 Lately, HAKI knives modified from existing surgical instruments are used in the treatment of trigger finger, and the surgery is guided through ultrasound for safety. 7 8 9

**Fig. 1:**
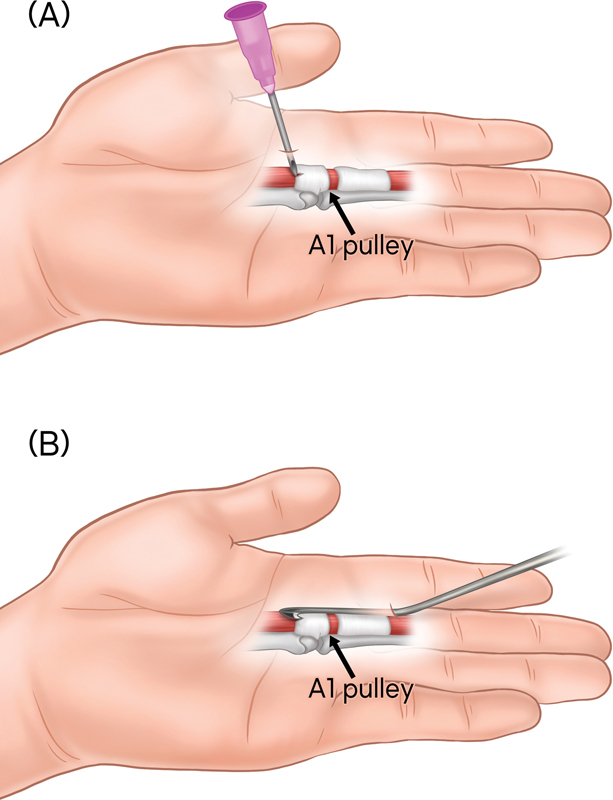
Schematic overview of percutaneous A1 pulley release procedure. ( **A** ) In the case of release using a needle, a vertical approach is made adjacent to the palmar crease. The tip of the needle is utilized like a knife to release the A1 pulley. ( **B** ) When employing a HAKI knife for release, the approach is made distally from the level of the proximal phalanx base. The hook portion of the knife is used to longitudinally incise the A1 pulley from proximal to distal. A1 pulley, first annular pulley.

The procedure is as follows: After administering local anesthesia to the digit affected by trigger finger, a needle is inserted just distal to the flexor crease where the A1 pulley is located. The bevel of the needle is oriented longitudinally with the tendon, and a longitudinal release of the A1 pulley is performed. 3 The tip of the needle can be utilized as a blade, or a needle knife-type instrument can also be employed 10 ( Fig. 1A ).

In cases using HAKI knife-type instrument, the approach is made distally from the base of the proximal phalanx. The blade is designed to be on the inner side and features a hook-shaped end. This allows for the pulley to be divided longitudinally from proximal to distal after the hook is positioned beneath the proximal margin 9 ( Fig. 1B ).

Both procedures are known to be safely performed with sonographic assistance, and their advantages have been highlighted in various publications. 1 These methods are known to be feasible as office procedures in outpatient clinics. 2 3 6 However, regardless of the simplicity compared to open release, the anatomical importance and requisite caution cannot be overstated. A heightened level of anatomical awareness is critical during the procedure. According to Aksoy and Sir, there is a known risk of damaging major structures, including nerve and tendon injuries, due to incautious execution at the site of the operation. 11

In this study, we aim to provide additional insights into complications that can arise from infections at specific anatomical locations in percutaneous procedures. We will achieve this through the reporting and analysis of the patient cases described subsequently.

## Cases

### Case 1

**Fig. 2:**
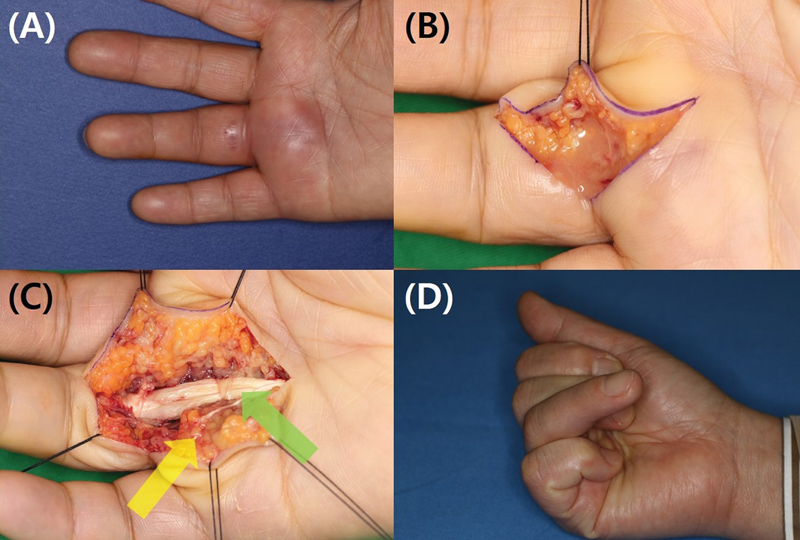
A 53-year-old woman with suppurative tenosynovitis after percutaneous A1 pulley release using a HAKI knife. ( **A** ) The patient presented with swelling and pain. ( **B** ) Clinical evaluation revealed inflammation accompanied by pus. ( **C** ) Surgical exploration identified additional injury to the flexor tendon (yellow arrow) and insufficient A1 pulley release (green arrow). ( **D** ) Ultimately, the patient developed a sequela characterized by adhesion of the flexor tendon, leading to functional limitations. A1 pulley, first annular pulley.

A 53-year-old female patient underwent sono-guided percutaneous A1 pulley release using a HAKI knife at a local clinic 1 month before visiting our hospital. Two weeks after the initial surgery, swelling and pain were observed ( Fig. 2A ). The patient visited our hospital and underwent Incision and Drainage (I&D). During this procedure, pus was observed, and both insufficient A1 pulley release and additional injury to the flexor tendon were found ( Fig. 2B, C ). Following a 2-week course of intravenous antibiotics, the patient exhibited an improvement in the inflammatory condition and was subsequently discharged. Nevertheless, a final limitation in the range of motion was noted as a sequela ( Fig. 2D ).

### Case 2

**Fig. 3:**
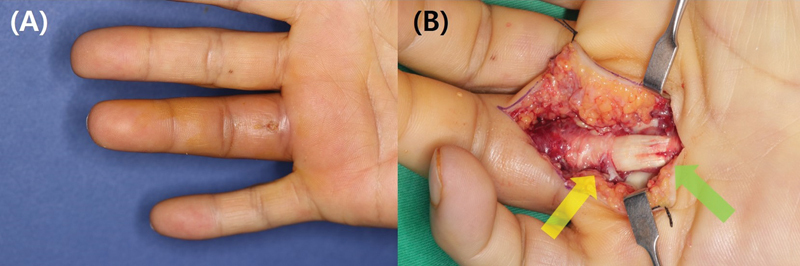
A 46-year-old man with suppurative tenosynovitis after percutaneous A1 pulley release using a HAKI knife. ( **A** ) The patient visited due to swelling and pain that occurred 1 week after undergoing the procedure. ( **B** ) During the exploration, it was confirmed that the patient had both a flexor tendon injury (green arrow) and an accompanying A2 pulley injury (yellow arrow). A1 pulley, first annular pulley; A2 pulley, second annular pulley.

A 46-year-old male visited our hospital 2 weeks subsequent to receiving a sono-guided percutaneous A1 pulley release employing a HAKI knife. The patient manifested symptoms of swelling and pain 1 week postoperatively ( Fig. 3A ). Given the clinical suspicion of bacterial infection, immediate surgical intervention was warranted, and I&D was executed. During the surgical exploration, it was ascertained that the A1 pulley had been adequately released. Nevertheless, concomitant longitudinal injuries to the flexor tendon and compromise of the second annular pulley (A2 pulley) were identified ( Fig. 3B ).

### Case 3

**Fig. 4:**
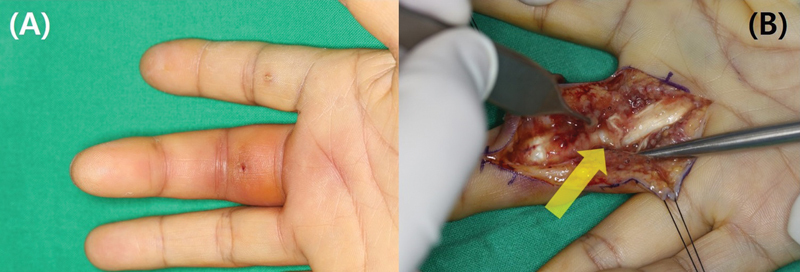
A 54-year-old woman with suppurative tenosynovitis after percutaneous A1 pulley release using a HAKI knife. ( **A** ) The patient visited due to swelling and pain that occurred 1 week after undergoing the procedure. ( **B** ) During the exploration, severe inflammation in the surrounding area and an accompanying A2 pulley injury were identified (yellow arrow). A1 pulley, first annular pulley; A2 pulley, second annular pulley.

A 54-year-old female patient presented to our institution 12 days subsequent to receiving a percutaneous A1 pulley release with a HAKI knife at the same local clinic as the previously described patient. One week postoperatively, the patient manifested infectious complications and sought medical care at our facility ( Fig. 4A ). Immediate surgical intervention was undertaken, and during the I&D procedure, injury to the A2 pulley was identified ( Fig. 4B ). Owing to the persistence of the infectious presentation, additional I&D was performed. After a 38-day inpatient stay complemented by antibiotic therapy, the patient demonstrated an improvement in the inflammatory condition and was subsequently discharged.

### Case 4

**Fig. 5:**
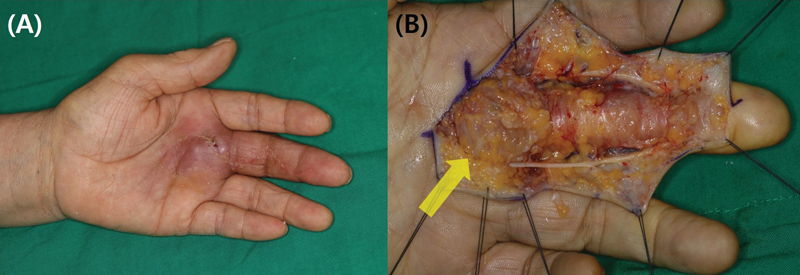
A 82-year-old woman with chronic inflammation after percutaneous A1 pulley release using a needle. ( **A** ) The patient had undergone ongoing incision and drainage at another hospital and experienced a recurrence of the infection after wound closure. Swelling accompanied by pus was present at the palmar crease level. ( **B** ) During the exploration, chronic inflammation resulted in granulomatous inflammation and tissue hypertrophy at the A1 pulley. A1 pulley, first annular pulley.

An 82-year-old female patient presented to our institution 4 months subsequent to undergoing a blind percutaneous A1 pulley release employing needles at a local clinic. Twelve days postoperatively, the patient manifested symptoms of swelling and pain. Despite receiving I&D at an alternative medical facility, the patient experienced a recurrence of infection and subsequently sought care at our hospital ( Fig. 5A ). Immediate surgical exploration was executed. During this intervention, excessive inflammatory granulation tissue on A1 pulley was identified ( Fig. 5B ). Following a 5-week regimen of intravenous antibiotics, the patient demonstrated an improvement in the inflammatory condition and was subsequently discharged.

### Case 5

**Fig. 6:**
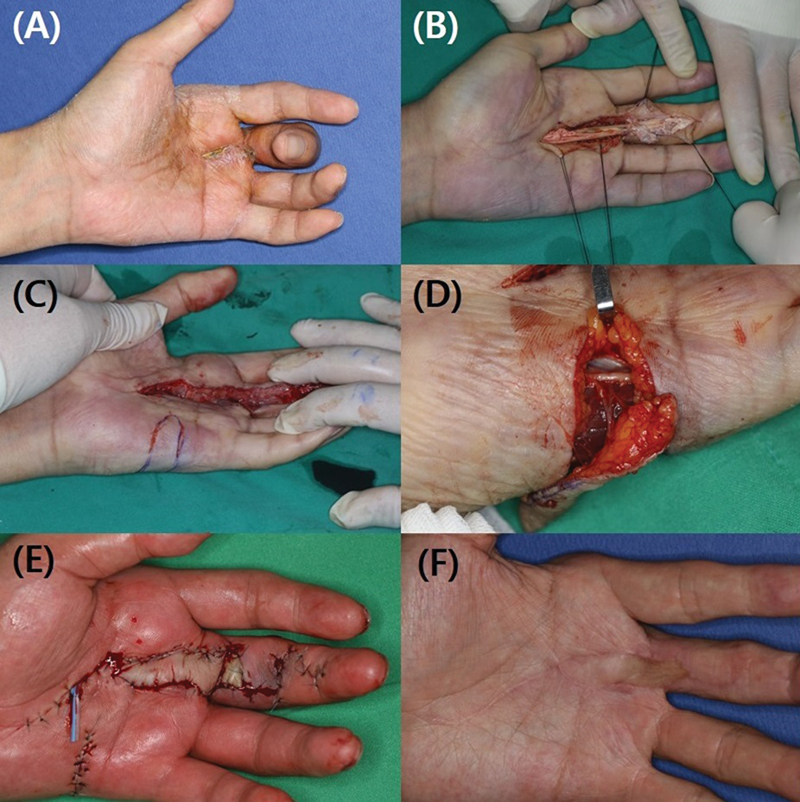
A 45-year-old man with soft tissue necrosis after percutaneous A1 pulley release using a needle. ( **A** ) The patient presented to our facility with tendon exposure due to soft tissue necrosis, despite undergoing repeated incision and drainage at another hospital following the development of suppurative tenosynovitis after a percutaneous A1 pulley release. ( **B** ) Inflammation was confirmed along the flexor digitorum profundus up to the proximal interphalangeal joint level. ( **C, D** ) A hypothenar perforator free flap was elevated to cover the soft tissue defect that occurred after debridement. ( **E** ) Flap insetting was performed. ( **F** ) Follow-up photographs taken 1 year later. A1 pulley, first annular pulley.

A 45-year-old male patient presented to our institution 2 months subsequent to undergoing a blind percutaneous A1 pulley release employing needles. Following the initial procedure, the patient manifested symptoms of infection. Despite undergoing three attempts at debridement and flexor tendon reconstruction at an alternative medical facility, the patient developed soft tissue necrosis and consequently sought care at our hospital ( Fig. 6A ). During surgical exploration, signs of inflammation were observed extending to the level of the proximal interphalangeal joint in the flexor digitorum profundus, and a longitudinal soft tissue defect was evident postdebridement ( Fig. 6B ). To address the tissue deficit, a hypothenar perforator free flap based on the fourth common digital artery perforator was executed 12 ( Fig. 6C–E ). Following a 2-week regimen of intravenous antibiotics, the patient was discharged. Long-term follow-up revealed no issues concerning flap coverage ( Fig. 6F ); however, an additional tendon transfer was necessitated to restore the range of motion.

### Case 6

**Fig. 7:**
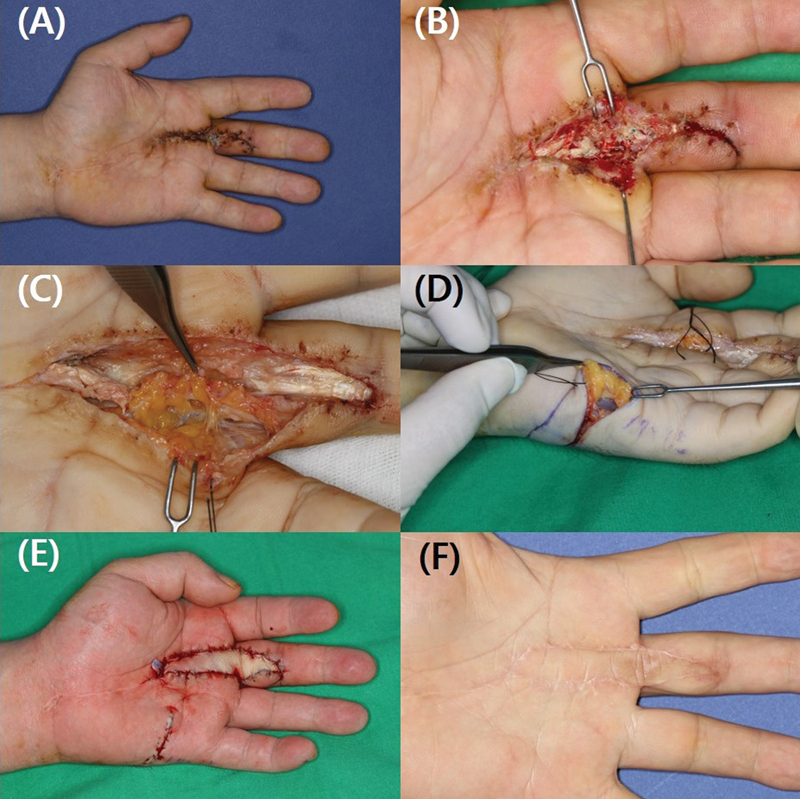
A 57-year-old man with soft tissue necrosis after percutaneous A1 pulley release using a needle. ( **A, B** ) The patient had received a procedure 2 years ago but continued to show signs of chronic inflammation despite undergoing repeated incision and drainage, as well as flexor tendon reconstruction at different hospitals due to recurring infectious complications, including tendon ligament infections. ( **C** ) Chronic infection-induced inflammation was observed in the surrounding areas. ( **D, E** ) After performing debridement, a hypothenar perforator free flap was used for defect coverage. ( **F** ) Follow-up photographs taken 1 year later. A1 pulley, first annular pulley.

A 57-year-old male patient presented to our institution 15 months after undergoing a sono-guided percutaneous A1 pulley release using needles at a local clinic. One week postoperatively, signs of infection emerged, leading to multiple I&D procedures at different hospitals. During these procedures, tendon necrosis occurred, necessitating attempted reconstruction. Despite these efforts, the site continued to exhibit persistent necrosis and inflammation, culminating in the patient's transfer to our hospital ( Fig. 7A ). Chronic inflammation involving the flexor digitorum profundus mandated extensive debridement ( Fig. 7B, C ). A hypothenar perforator free flap, based on the fourth common digital artery perforator, was performed to address the ensuing soft tissue defect 12 ( Fig. 7D, E ). After a 3-week course of inpatient treatment, the patient was discharged. Long-term follow-up showed no signs of inflammation in the flap coverage area ( Fig. 7F ); however, a secondary tendon reconstruction was subsequently required.

## Discussion

Previous studies have mainly reported that percutaneous A1 pulley release is safe and effective with more advantages as compared to the conventional open technique. Many cadaveric studies also revealed that percutaneous release did not lead to adjacent organ injury and was safe and accurate, 1 2 3 and other several groups also recommended percutaneous release over conventional open resection. 7 While percutaneous release was not associated with adjacent organ injuries in any other studies, 8 it has been observed to cause small surgical wounds with fewer complications (like bowstrings) as compared to open technique. 13 In randomized controlled trials, where the trigger finger recurrence rate after percutaneous release was not significantly different from that after open release, percutaneous release showed significantly better results of rehabilitation than open release. 4 14 Moreover, using sonograph to guide the instruments can reduce the risk of incomplete release as well as other complications, enabling more precise procedures. 15 However, recent research has reported that percutaneous pulley release can occasionally cause flexor tendon injury, ranging from minor longitudinal scoring to rare cases of delayed tendon rupture. 8 11

Anatomically, a flexor sheath is a double-walled structure that surrounds the flexor tendon to form a closed continuous synovial system. 16 Infection by bacterial inoculations in this space induces bacterial overgrowth along the inner space of the synovium which increases pressure due to fluid collection, causing ischemia and septic necrosis. 17 Here, we observed that infections may occur in the flexor sheath space after percutaneous A1 pulley release. Even though the surgeries were guided with ultrasound, the procedures were incomplete and led to hidden organ injuries.

Despite the limited patient population included in this study and the difficulty in directly extrapolating the rate of complications arising postprocedure in local clinics, this study aims to discuss the characteristics of the complications observed in the six patients included. By analyzing these cases, we intend to provide insights into the nature of such complications as well as directions for future research and preventive measures.

From another perspective, this study serves to identify infectious complications of percutaneous A1 pulley release, which were unexplored in cadaveric studies that had previously emphasized the procedure's safety. 1 8 18 Notably, skin flora was found in the wound culture, suggesting that the infectious complications may be directly attributable to the procedure itself ( Table 1 ). This adds critical nuance to our understanding of the risks involved, especially in the context of real-world applications as opposed to cadaveric models.

**Table 1:** Demographic information of patients ( *n* = 6)

Case No.	Age (y)	Sex	Side/finger	Approach site	Sono-guided (Y/N)	History	Intervals between procedure and infection (d; average = 8.5)	Antibiotic treatment period (d; average = 23.3)	Wound culture
1	53	F	R/ring	Finger	Y	DM	14	14	MSSA
2	46	M	R/ring	Finger	Y	None	7	12	MRSA
3	54	F	R/middle	Finger	Y	None	8	38	MRSA
4	82	F	L/middle	Palm	N	None	11	39	MRSA
5	45	M	L/middle	Palm	N	None	3	16	MRSA
6	57	M	L/middle	Palm	Y	DM	8	21	MRSE

Abbreviations: F, female; L, left hand; M, male; R, right hand.

One potential factor contributing to the onset and progression of the infection could be a delayed diagnosis. This is substantiated by the fact that in all admitted patients, at least 1 week elapsed postprocedure before the initiation of infection assessment and treatment ( Table 1 ). Given that percutaneous A1 pulley release is predominantly performed in local clinics, as evidenced by the patient cohort in our study, there exists an elevated risk that diagnoses may be deferred during outpatient care. This simultaneously implies that timely intervention and treatment may not be realized, thereby increasing the complexity and severity of the infection and its associated complications.

Also, it is interesting to note that the complications showed different trends depending on the type of surgical instrument used ( Table 2 ). The hook-type HAKI knife has the structural advantage of an easily releasing A1 pulley. However, our findings implicated that the hook structure may have caused blind injuries in adjacent organs such as the flexor tendon and A2 pulley despite the surgeries being guided with ultrasound. In addition, since HAKI knives are made of metal, they can be reused after sterilization. As the knife is structurally concave, foreign materials may not have been cleaned completely, causing possible bacterial infections.

**Table 2:** Complications of the patients

Case No.	Instrument	Insufficient A1 Pulley release (Y/N)	Additional injuries	Skin necrosis
1	HAKI knife	Y	Tendon	N
2	HAKI knife	N	Tendon, A2 pulley	N
3	HAKI knife	N	A2 pulley	N
4	Needle	Y	None	N
5	Needle	Unknown	Unknown	Y
6	Needle	Unknown	Unknown	Y

Abbreviation: A1, first annular pulley.

In contrast, the lumen structure of needles allows direct bacterial inoculation into the synovium. As a result, the unopened pyogenic tenosynovitis may have rapidly progressed inside the synovium, leading to serious complications such as soft tissue necrosis (Cases 5 and 6). For both the patients, defect coverage using the fourth common digital artery perforator, a glabrous skin free flap characteristically similar to the defect site, was confirmed to be a useful reconstructive method. 12

As described, various risk factors may be present. However, studies mainly report percutaneous release as a safe and simple procedure despite possible risks of serious negative outcomes. As a result, the procedure is sometimes conducted by nonprofessionals, which must be prevented. Anatomical misunderstandings can lead to an insufficient release of the A1 pulley and cause additional injuries and potential infection.

Therefore, based on our findings, we emphasize the importance of aseptic procedures for the surgery and provide the following recommendations:

- As percutaneous A1 pulley release is a blind procedure, it requires specific techniques and experience for sonography that provides an additional field of view. The procedure was performed after sufficient experience with sonographic image manipulation.
- It is recommended to use prophylactic antibiotics prior to percutaneous A1 pulley release. Underlying diseases must be checked for. If the patient has prior infection risks due to diabetes and autoimmune diseases, antibiotics must be used.
- The surgeon is recommended to have a complete understanding of the surgical instruments such as needle and knife to enable precise imaging interpretation and sufficient experience through cadaver dissection prior to performing the surgery in clinical practice. 1 18
- Minor tendon injury or insufficient pulley release from the procedure will not cause tendon problems or trigger relapse. However, these may cause serious complications such as infection. Therefore, surgeons who are not confident in the procedure must consider open release.
- Monitoring, including active physical examination, is required after the procedure is performed at local clinics. 17 The patients must be notified that close observation is required for up to 10 days after the procedure, and patients need to be actively treated for any suspected infection.
